# Prospects for the computational humanization of antibodies and nanobodies

**DOI:** 10.3389/fimmu.2024.1399438

**Published:** 2024-05-15

**Authors:** Gemma L. Gordon, Matthew I. J. Raybould, Ashley Wong, Charlotte M. Deane

**Affiliations:** Oxford Protein Informatics Group, Department of Statistics, University of Oxford, Oxford, United Kingdom

**Keywords:** humanization, humanness, antibody, nanobody, computational, therapeutics

## Abstract

To be viable therapeutics, antibodies must be tolerated by the human immune system. Rational approaches to reduce the risk of unwanted immunogenicity involve maximizing the ‘humanness’ of the candidate drug. However, despite the emergence of new discovery technologies, many of which start from entirely human gene fragments, most antibody therapeutics continue to be derived from non-human sources with concomitant humanization to increase their human compatibility. Early experimental humanization strategies that focus on CDR loop grafting onto human frameworks have been critical to the dominance of this discovery route but do not consider the context of each antibody sequence, impacting their success rate. Other challenges include the simultaneous optimization of other drug-like properties alongside humanness and the humanization of fundamentally non-human modalities such as nanobodies. Significant efforts have been made to develop *in silico* methodologies able to address these issues, most recently incorporating machine learning techniques. Here, we outline these recent advancements in antibody and nanobody humanization, focusing on computational strategies that make use of the increasing volume of sequence and structural data available and the validation of these tools. We highlight that structural distinctions between antibodies and nanobodies make the application of antibody-focused *in silico* tools to nanobody humanization non-trivial. Furthermore, we discuss the effects of humanizing mutations on other essential drug-like properties such as binding affinity and developability, and methods that aim to tackle this multi-parameter optimization problem.

## Introduction

An immunogenic response against a therapeutic antibody, including the production of anti-drug antibodies (ADAs), can reduce drug efficacy and negatively impact the patient (1). It is critical that this risk is minimized ahead of a drug entering human trials.

The earliest experimental approaches to discover target-specific therapeutic antibodies involved inoculating a non-human organism with the antigen of interest to raise complementary antibodies. If these antibody clones were to be injected directly into a human patient, an anti-drug immune response would be a very likely outcome, as was found to be the case for the first monoclonal antibody therapy, Muromonab (2).

**Figure 1:**
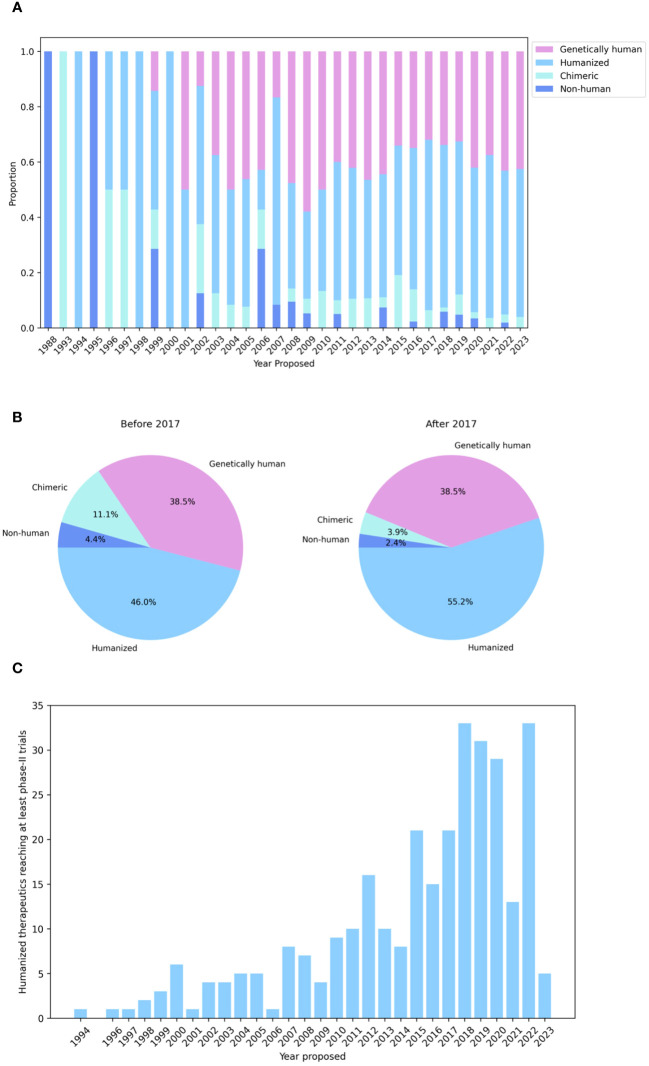
Humanization is the leading method for generating antibody therapeutics compatible with the human immune system. **(A)** Genetics of all WHO-recognized antibody- and nanobody-derived therapeutics included in the Thera-SAbDab database (4), by year of proposed International Nonproprietary Name (INN). **(B)** Developmental origins of therapeutics with a proposed INN before and after 2017 show that humanization is still the predominant means of generating antibodies for therapeutic use. **(C)** Cumulative number of humanized therapeutics recorded in Thera-SAbDab reaching at least phase-II clinical trials, by year of proposed INN. Statistics for recent therapeutics are likely to increase with time.

Logic would suggest that the immunogenicity of a non-human biologic might be mitigated by somehow increasing its ‘humanness’, loosely defined as similarity to antibodies raised naturally and tolerated by healthy human immune systems. This theory has sparked the development of an array of technologies to ‘humanize’ non-human antibodies. Though numerous techniques to discover genetically human antibodies have also been developed, such as constructing phage/yeast display libraries, using transgenic mice, or isolating antibodies directly from convalescent humans (3), it is striking that most recent therapeutics still derive from non-human organism inoculation followed by humanization (**Figure 1**; 4).

Therefore, resolving outstanding challenges within this long-established field remains highly relevant. For example, grafting strategies that load non-human complementary-determining regions (CDRs) onto human framework scaffolds are unsuccessful when the variable loops play a role in immunogenicity and can compromise other key developability properties. Increasingly, computational approaches are offering a route toward identifying and mitigating factors contributing to immunogenicity, as well as enabling the simultaneous optimization of other drug-like properties alongside humanness (5).

Additional challenges are posed by nanobodies, a fundamentally non-human modality deriving from camelids (VHHs) or cartilaginous fish (VNARs) (6, 7), which are emerging as a promising therapeutic format. Their smaller size facilitates expression, improves tumor penetration, and increases solubility, while maintaining comparable binding affinity to conventional antibodies (8–13). However, structural differences between antibodies and nanobodies affect how they interact with their antigens (14). Therefore, it is likely that many humanization protocols designed for conventional antibodies, particularly computational tools, are not immediately applicable to nanobodies.

Previous reviews cover experimental humanization approaches for conventional antibodies, such as CDR grafting or resurfacing (15, 16). More recent publications address the use of machine learning in the antibody discovery field, including *in silico* methods for humanization, humanness scoring and immunogenicity prediction within the broader scope of antibody design (5, 17–22), and draw attention to the need for multi-parameter optimization (23, 24). Focus is largely placed on the development of conventional antibodies as opposed to alternative formats, though the need for work on nanobodies is highlighted in Norman et al. (17). Rossotti et al. (7) consider nanobody humanization but focus on experimental rather than computational methods.

In this review, we start by introducing early approaches to humanization and, more broadly, how the immunogenicity of an antibody can be quantified. We cover computational methodologies designed to measure humanness and direct humanization (**Table 1**), including the degree of evidence to support the efficacy of each protocol and their potential applicability to nanobodies. Finally, we place humanization in the wider context of antibody design and multi-parameter optimization, highlighting the need for high-quality, unbiased data to overcome the challenges that persist in this field.

**Table 1:** Summary of available computational methods for humanness scoring and humanization, in order of reference in this review.

Authors	Year	Name	Web server	Reference
Abhinandan and Martin	2007	H-score	http://www.bioinf.org.uk/abs/shab/	(25)
Thullier et al.	2010	G-score	N/A	(26)
Gao et al.	2013	T20 score	https://sam.curiaglobal.com/t20/	(27)
Lazar et al.	2007	Human String Content	N/A	(28)
Seeliger	2013	N/A	N/A	(29)
Clavero-Álvarez et al.	2018	MG-score	N/A	(30)
Schmitz et al.	2020	PGSSM score	N/A	(31)
Marks et al.	2021	Hu-mAb	https://opig.stats.ox.ac.uk/webapps/sabdab-sabpred/sabpred/humab	(32)
Prihoda et al.	2022	BioPhi	https://biophi.dichlab.org/	(33)
Uçar et al.	2023	SelfPAD	N/A	(34)
Zou et al.	2023	PLAN	N/A	(35)
Wollacott et al.	2019	N/A	N/A	(36)
Vashchenko et al.	2022	AbBERT	N/A	(37)
Choi et al.	2015, 2016	CoDAH	N/A	(38, 39)
Hsieh et al.	2022	N/A	N/A	(40)
Tennenhouse et al.	2023	CUMAb	https://cumab.weizmann.ac.il/step/cumab-terms/	(41)
Sang et al.	2022	Llamanade	http://www.llamanade.app	(42)
Ramon et al.	2023	AbNatiV	www-cohsoftware.ch.cam.ac.uk/index.php/abnativ	(43)

The expanded form for ‘N/A’ would be ‘Not available’.

## Experimental approaches to humanization

**Figure 2:**
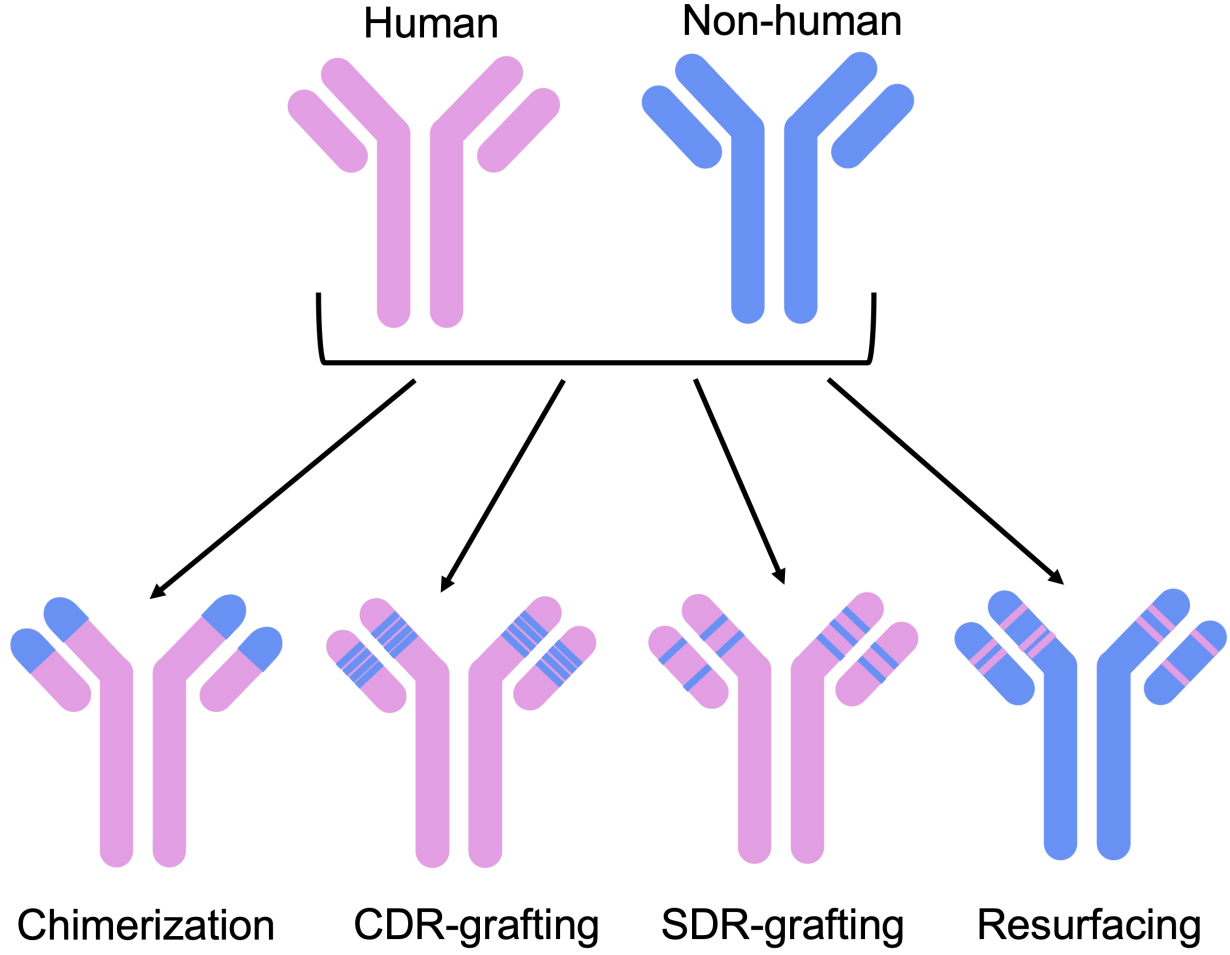
Humanization methods have evolved from initial chimerization approaches, aiming to reduce non-humanness without compromising functionality. These include CDR-grafting, where non-human CDR loops are grafted on to a human framework, SDR-grafting, where only the binding residues of the CDRs are grafted on to the framework, and resurfacing, where exposed non-human residues are replaced by human ones.

Experimental methods seek to reduce the immunogenicity of an antibody by increasing the human portion of its variable domains (**Figure 2**). The first broadly applicable strategy to achieve this was chimerization, the grafting of a non-human variable region onto human constant domains (44).

Further increases in the humanness of the antibody sequence were made possible by humanization techniques such as only grafting the CDRs (complementarity-determining regions) of a non-human antibody onto a human framework region (45). In theory, this approach serves to preserve binding activity, since the CDR loops tend to form most of the binding site, alongside the structural stability provided by a native framework. However, CDR grafting has often been found to require additional changes to the framework, such as back-mutations to the original non-human residue, to improve or even rescue binding (46).

Reducing the number of non-human residues even further, structures of antibody-antigen complexes have been used to determine the binding site residues of a non-human antibody and use only these from the non-human CDR loops (specificity-determining region (SDR) grafting) (47, 48). Similarly, Apgar et al. (2016) (49) developed a method to identify important binding residues and reduce immunogenic residues in non-human CDR loops, after CDR grafting.

To preserve favorable properties of the non-human antibody, humanization has also been carried out using the most closely related human germline sequences or homologous framework regions as a template (16). Alternative approaches include framework shuffling (50), where a set of framework regions representative of all human germline genes were iteratively combined with the CDR loops of a non-human antibody and their binding activity assessed. Another method, resurfacing, preserves the non-human frameworks: only surface-exposed residues which differ from human are replaced on the basis that buried residues will not impact the level of immunogenicity (51, 52).

Collectively, these experimental strategies have been widely integrated into pharmaceutical companies’ preclinical therapeutic development pipelines and have yielded developable molecules, as evidenced by the ever-growing number of humanized antibodies progressing through first-in-human clinical trials (**Figure 1C**). Nevertheless, success remains clone dependent, and efficiencies could certainly be found in directing the humanization process, in which amino acid mutations are frequently introduced *via* trial-and-error. The concurrent integration of computational methods, which attempt to learn sequence and structural features of antibodies to predict the impact of modifications, promises to lead to more rational and efficacious humanization.

## Quantifying the “humanness” of an antibody and its utility for computational humanization

While the most direct measure of immunogenicity is the quantification of anti-drug antibodies (ADAs), T cells, or inflammatory cytokines raised upon injection of the drug into an organism, this carries an inherent safety risk, and so indicative assays are used during early-stage development.

Computational methods have gained traction in the quantification of the humanness of a sequence, hypothesized as a proxy metric for the likelihood of immunogenicity due to the evident success of experimental grafting strategies.

An early example of a computational humanness score is the H-score (25), which uses pairwise sequence identity to distinguish between human and non-human variable regions. The G-score was later derived from the H-score to account for the effects of the size of the corresponding human germline family (26). This was further refined to the T20 metric by Gao et al. (27), who incorporate a BLAST search, scoring by taking the average percentage sequence identity of the top 20 matching sequences. Lazar et al. (28) break sequences down into 9-mers, proposing the Human String Content (HSC) score, which is calculated by comparing the sequence identity of each 9-mer frame in a sequence to human germline sequences. Together, these methods were validated simply by demonstrating distinct distributions of humanness scores between human and non-human species. For example, Abhinandan and Martin (25) showed a separation between the mean ‘raw humanness’ scores and Z-scores of human and murine antibodies. Improving on this, the T20 score was shown to distinguish between chimeric, humanized, and human sequences with greater specificity than the H-score or G-score on a set of 98 therapeutic antibodies. Further to this, Gao et al. present perhaps the first quantitative benchmark between an *in silico* humanness metric and a direct measure of immunogenicity, having demonstrated on a dataset of 65 therapeutic antibodies that higher (more human) T20 scores correlate, albeit weakly (Pearson’s correlation of 0.21), with lower ADA abundance.

To capture higher-order relationships between amino acid residues, Seeliger (29) designed a heuristic scoring function to distinguish between human and murine antibodies, using commonly occurring mutations as a fingerprint for the species. Like previous work, their humanness scores show a small overlap between murine and human antibodies, given the shared sequence identity of the two species. Using a statistical inference method to account for correlations between residue pairs at different positions, Clavero-Álvarez et al. (30) created an ‘MG-Score’ metric, finding that their approach outperforms the T20 score at differentiating between human and murine sequences, although these methods are equally predictive for the smaller therapeutic datasets tested.

Schmitz et al. (31) introduce a position- and gene-specific scoring matrix (PGSSM) metric, which uniquely uses single nucleotide frequencies to measure the similarity of a sequence to a human antibody repertoire. The authors found that human sequences scored significantly higher than other species including non-human primates, using their metric. In a broader application to antibody developability, Petersen et al. (53) use a position-specific scoring matrix representing antibody repertoire data to predict high-frequency framework mutations that could improve therapeutic properties.

Although sequence identity indicates how closely related a human antibody and non-human antibody are, it may not be the most informative metric for the purpose of humanization. More recent strategies such as those from Seeliger (29) and Clavero-Álvarez et al. (30), or those using scoring matrices could offer an advantage in that they provide insight into higher-order features: it is possible to determine which residues are most important in contributing to humanness versus which are common across species. This in turn can be used to inform the choice of mutations for humanization.

Naturally, the greater availability of sequence data (54, 55) has made *in silico* humanness scoring and humanization amenable to machine-learning methods. Hu-mAb (32) approaches this problem as a classification task, using random forest models to distinguish human from non-human sequences, achieving ROC AUC values across all classification models of 1 or close to 1. Similarly to Gao et al. (27), the authors curated an ADA benchmark dataset, this time for an enhanced set of 217 therapeutics, demonstrating a stronger correlation between higher Hu-mAb humanness scores and lower immunogenicity. They also demonstrated that therapeutics with the most severe ADAs frequently had Hu-mAb scores below 0.9.

The same dataset of 217 therapeutics was used for evaluation by Prihoda et al. (33) and Uçar et al. (34). Uçar et al. used a contrastive-learning model to predict humanness, leveraging a large body of patent data, while Prihoda et al. present the BioPhi platform, developed using Transformer architecture. BioPhi consists of tools for both humanization (Sapiens) and humanness scoring (OASis), the latter derived from the use of 9-mer peptides in the HSC scoring method (28). The platform can operate at different levels of stringency: at a medium level (a peptide is defined as human if it is found in at least 50% of subjects), OASis outperformed other humanness scoring methods, such as the aforementioned T20 and MG-scores, with a ROC AUC of 0.966, though it was comparable to the PGSSM metric from Schmitz et al. (31). Authors find a similar correlation between their humanness scores and the ADA responses to the therapeutic set, though do not outperform Hu-mAb (achieving a Pearson correlation of 0.28, compared to Hu-mAb’s 0.34).

Marks et al. (2021) further validate Hu-mAb by comparing their computationally suggested mutations against a ground truth of experimentally chosen mutations for a set of 25 humanized therapeutics, all of which showed reduced immunogenicity upon humanization. Overall, they found Hu-mAb more efficiently suggested mutations that overlapped considerably with the mutations made experimentally (77%, or 85% when including residues of similar types). A similar approach is taken by Prihoda et al., (33) and Zou et al. (35), who adopt the BioPhi OASis humanness scoring method in their proposed humanization approach, which makes use of protein language models alongside a k-nearest-neighbors method to optimally select residues for humanization. Prihoda et al. expand their test set to include 152 humanized antibodies and putative parental sequences.

A metric often used to validate humanization is the change in humanness score after mutation, where scoring methods are developed using a dataset of human antibodies as a benchmark. Assuming a reliable and valid negative correlation between this humanness metric and immunogenicity, an increase in the humanness score of a putatively humanized sequence before and after mutation would indicate that humanization has been successful. This is adopted by Marks et al. (32), Zou et al. (35), Wollacott et al. (35, 36) and Vashchenko et al. (37). Long short-term memory (LSTM) models trained by Wollacott et al. on natural antibodies can be used to quantify the nativeness of antibody sequences and to select templates for humanization, based on changes in their LSTM humanness score. AbBERT (37) is an attention-based Transformer trained on 20 million unpaired sequences from OAS (55) primarily to determine humanness (for which greater humanness scores are demonstrated for human sequences over humanized and murine sequences), but embeddings can be used to optimize *in silico* antibody design. Vashchenko et al. (37) are additionally conscious of the balance between humanness and optimization of other antibody properties such as stability: authors test for expression levels, finding that in general, more human antibodies were better expressed.

Structure-guided methods for computational humanization can make use of means of validation that cannot be adopted by methods built on sequence data, given the intrinsic differences in the nature of the data used. For example, Choi et al. (38, 39), Hsieh et al. (40) and Tennenhouse et al. (41) all adopt binding affinity as their main indicator for the success of their humanization, specifically whether the affinities of their proposed humanized variants are comparable to their parental antibody.

Choi et al. present CoDAH (38, 39), which aims to produce designs that increase humanness without disrupting stability. Hsieh et al. (40) implement homology modeling and molecular dynamics simulations to compare murine and humanized CDR structures, as changes in these residues impact the resulting binding affinity. Given the effect that CDR-grafting can have on stability and affinity, Tennenhouse et al. (41) present CUMAb, which seeks to simulate this CDR-grafting onto human frameworks and select designs using an energy-based ranking. Computationally, this can be carried out at much higher throughput and CUMAb searches a more diverse structural space, testing non-homologous frameworks as well as homologous templates that are the default for CDR-grafting.

The combination of close similarity between a proposed humanized variant and human antibodies, and maintaining binding affinity, imply that humanization has successfully increased humanness without compromising native features which may contribute to binding or other favorable properties of the therapeutic. However, to evaluate the performance of these tools more robustly, the reliability of the humanness scoring method must also be considered. Additionally, while maintaining affinity is undoubtedly important in the success of a humanized antibody, relying solely on affinity for the validation of these humanization methods will not fully encapsulate their capabilities.

Overall, these computational methods capture humanness in different ways, and each have their own merits as measurements, but vary as to what extent, and by what means, they are validated as predictors of immunogenicity. An increased humanness score, the most-used validation metric across all tools, does not *de facto* guarantee reduced immunogenicity. Humanness metrics which are validated against experimental data, such as those that exhibit a correlation to the incidence of ADAs across therapeutics, or whose values support experimental humanization decisions for a case study antibody that were proven to decrease immunogenicity, might reasonably be considered more robust predictors. Furthermore, it is important to consider that machine learning approaches, which are now abundant in this field, are reliant on the data that they are built upon: humanized antibodies generated by these models will harbor the inherent biases of the training set, which could limit their real-world applicability.

## Applicability of antibody humanization software to nanobodies

MAbs are significantly more established as biotherapeutics than nanobodies, meaning benchmark datasets are more readily available and so efforts thus far have been focused on developing *in silico* methods for conventional antibodies. For example, as illustrated above, a dataset of 217 therapeutics with ADA data has been used for validation of antibody-focused computational approaches. In comparison, to our knowledge, ADA data only exists for 10 nanobody therapeutics (**Table 2**, 56–73). This discrepancy extends to a differential availability of natural sequence data from which to build models. There are far more publicly available antibody repertoire data than nanobody repertoire data: at present, approximately 1.6 million nanobody sequences are deposited in the OAS database, compared to over 2 million paired and 2.4 billion unpaired antibody sequences (55).

**Table 2:** Nanobody therapeutics with available ADA data.

Name	Phase	Therapeutic (T)/Placebo (P)	Total participants	% Patients with ADA	Reference
2Rs15d	-	T	20	0	(56, 57)
	-	T	20	5	
ALX-0061	I/II	T	37	0	(58–61)
	II	T	250	41	
		P	62	52	
	IIb	T	187	31	
ALX-0081	I/Ib	T	64	0	(62–64)
	II	T	36	9	
	III	T	145	3	
ALX-0141	I	T	42	0	(65)
ALX-0171	I	T	60	0	(66, 67)
	IIb	T	135	34	
		P	39	26	
ALX-0761	I	T	33	30.3	(68)
		P	8	37.5	
ATN-103	I/II	T	266	3	(69–71)
M6495: Construct 579 (2F3*SO35GS linker093*SO linkerAlb11)	-	T	50	6	(72)
M6495: Construct 581 (2F3*SO35GS linker Alb11)	-	T	50	0	(72)
TAS266	I	T	4	75	(73)

Where applicable, dose-dependent figures have been aggregated for total participants and percentage of patients with ADAs.

**Figure 3:**
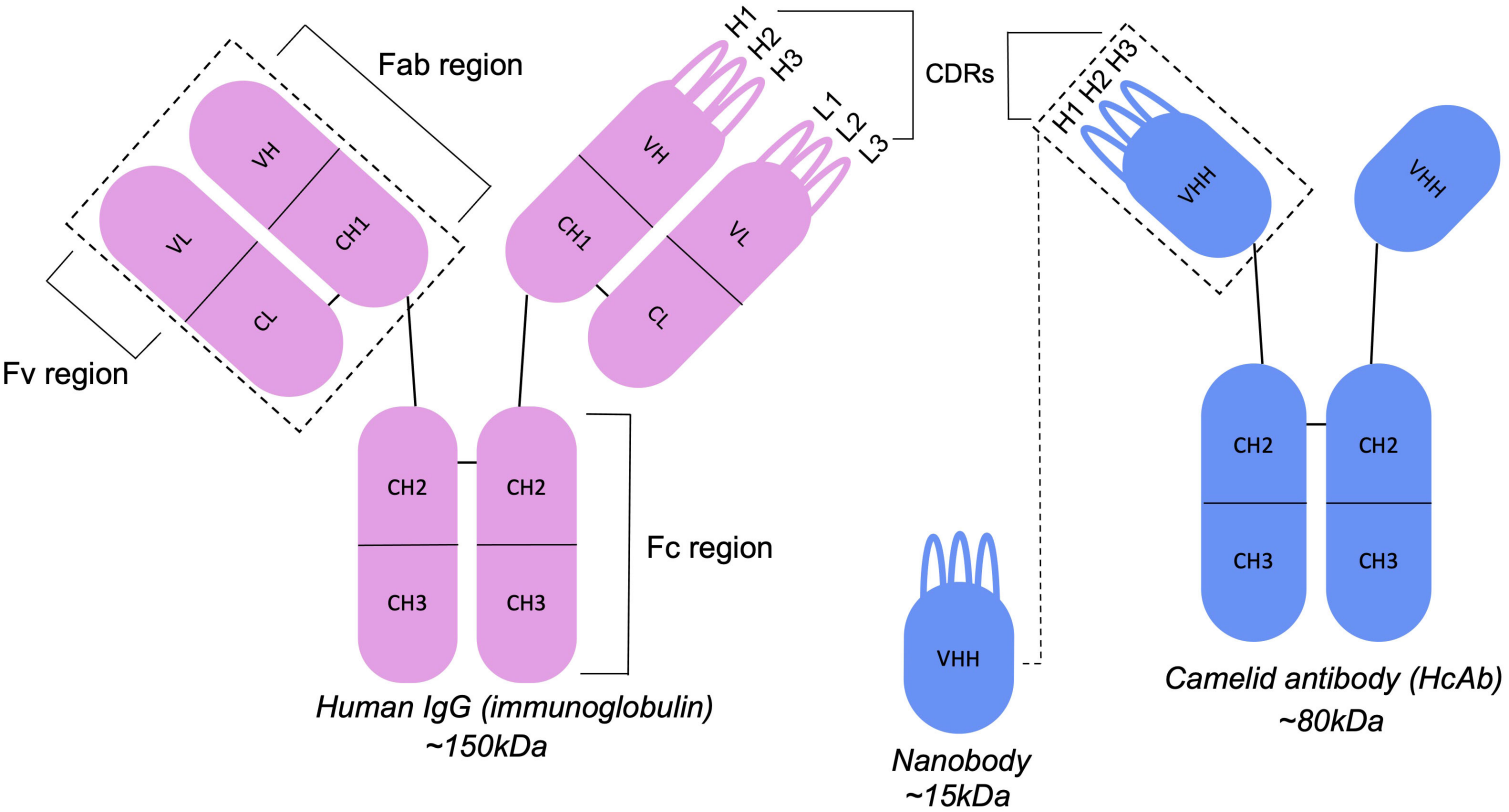
Antibodies and nanobodies differ in their structural features. Camelid nanobodies (VHHs) are derived from heavy-chain antibodies and lack the light-chain partner seen in conventional antibodies, exposing residues that would otherwise be buried at the VH-VL interface.

However, given the increasing application of nanobodies in the therapeutic space, future work developing humanization approaches should consider their applicability to other formats and modalities. General principles for the experimental humanization of nanobodies are akin to those for antibodies, such as CDR grafting and back-mutation, resurfacing and the use of germline sequences as templates (74). However, there are additional considerations to make since antibodies and nanobodies have distinct structural features (**Figure 3**). Vincke et al. (9) outline the humanization of a camelid nanobody (VHH) and observe the importance of residues in the FR2 region in contributing to binding affinity. These hallmark residues, located at an equivalent position to those at the VH-VL interface in antibodies, are usually hydrophilic and are hypothesized to contribute to the increased solubility of the single-domain antibody (8, 75). Since these residues are buried and hydrophobic in conventional antibodies, *in silico* humanization tools built for antibodies may suggest changes to these residues. Although this may increase their humanness, it may also diminish the favorable properties that nanobodies possess. Furthermore, this could decrease developability and increase immunogenicity, since exposed hydrophobic residues increase aggregation propensity (76–78).

In addition to their distinct hallmark residues, nanobodies more often incorporate framework residues into their paratopes (the antigen binding site) (12, 14, 79, 80). This is critical to consider in developing computational methods based on protocols such as CDR grafting, where it may be assumed that only the CDRs contribute to antigen-binding. As highlighted by Hummer and Deane (81), this is a pitfall encountered with CUMAb (41), where their approach to entirely replace the framework during *in silico* CDR-grafting may remove critical binding residues in nanobodies.

## Bespoke computational methods for nanobody humanization

Therefore, there is a need for computational methods that take the characteristic topology of nanobodies into account; the structure modeling software NanoBodyBuilder2, which outperforms AlphaFold2 by 0.55 Å over the CDR3 region, exemplifies the advantages of nanobody-specific tools (82). The development of humanization software dedicated to nanobodies is still a relatively new endeavor, the first being Llamanade (42). Sang et al. identify properties specific to Nbs by comparison with IgGs and use this as the basis for rational Nb humanization, avoiding humanization of residues that are integral to the nanobody physicochemical properties, such as highly conserved framework residues in the FR2 region and those which may contribute to the conformation of the CDR3 loop. This balance between nativeness (keeping residues critical to the unique structural properties of the nanobody) and humanness is also prioritized in AbNatiV (43), a deep-learning-based pipeline for nanobody (and antibody) humanization. Sequence data is used to train variational auto-encoders (VQ-VAE) models which quantify the similarity of a sequence to human VH or camelid VHH domains. This measure of nanobody or antibody nativeness is coupled with a sequence profile that can be used to inform engineering.

The Llamanade pipeline was assessed by calculating T20 scores (27) and conducting ELISA tests for 9 SARS-CoV-2 binders. The authors observed an increase in humanness scores, and, upon expression, 8 out of the 9 structures exhibited binding capabilities comparable to the original structures. The use of the T20 score in their evaluation invites confidence in their approach, given that Gao et al. (27) previously demonstrated some correlation between increased humanness and decreased immunogenicity. AbNatiV takes a similar approach to verify their humanness scoring, finding a negative correlation between humanness and the percentage of patients who developed ADAs for 216 therapeutic antibodies. Following Llamanade, humanization conducted by the AbNatiV pipeline is validated by the characterization of humanized variants of two nanobodies: Ramon et al. (43) find that their method improves or retains thermostability and binding compared to wild-type variants. Both works by Sang et al. (42) and Ramon et al. (43) are subject to the same difficulties in their use of humanness scoring and affinity as their primary means of validation, as was previously discussed. This is only exacerbated by the more restricted sequence and structural data available for nanobodies, which, as highlighted by Sang et al. (42), limits opportunities to quantify the uncertainty of proposed designs.

## Humanization within the multi-parameter optimization problem

All previously outlined computational approaches consider humanization in isolation, however, when designing an antibody therapeutic, it is important to be aware of the full landscape of properties that need to be optimized. Immunogenicity can originate not only from intrinsic humanness but also from developability factors, including solubility, aggregation likelihood, cross reactivity, and product heterogeneity deriving from sub-optimal chemical or thermal stability of the antibody (83–85). More broadly, the multi-parameter optimization problem may pertain to patient or treatment-related factors affecting therapeutic success. For example, a patient’s immune status, such as having a chronic infection or being depleted of B cells, might impact the likelihood of observing ADAs. The personalized combination of human leukocyte antigen (HLA) alleles in each patient influences the propensity of autologous helper T cells to recognize drug fragments, and therefore activate B lymphocytes toward the expression of ADAs (86); tools such as NetMHCIIpan-4.0 (87) can survey whether drugs contain peptide fragments able to be displayed by certain Class II HLA alleles. Meanwhile, more convenient routes of administration that increase the global accessibility of a medication, such as subcutaneous injection, typically require more concentrated doses that are more susceptible to molecular aggregation and thus the induction of ADAs.

The use of computation to simultaneously optimize or select antibodies (or nanobodies) with a general set of drug-like properties represents a compelling strategy toward reducing the failure rate of candidate therapeutics and accelerating the antibody design pipeline. Excitingly, such approaches are already coming to the fore. Makowski et al. (88) assess how machine learning can be used to co-optimize the affinity and specificity of Emibetuzumab through a joint reward function. Furthermore, Bachas et al. (89) use language models to predict binding affinity alongside a ‘naturalness’ metric. They show that this naturalness relates to developability and immunogenicity and, as such, optimizing both affinity and naturalness together using a genetic algorithm may help to solve this multi-parameter optimization problem. There are also increasing examples of work using machine-learning to generate new sequences using antibody libraries with favorable properties as a source of high-throughput data (90, 91). Arras et al. (92) developed a semi-synthetic method to generate humanized and developable nanobodies (VHH) from camelid immunization, with minimal need for further optimization. Camelid CDR3 loops are grafted onto humanized VHH backbone libraries with diverse combinations of camelid CDR1 and CDR2 loops. The library is then filtered for developability attributes and biophysical properties are tested experimentally. In subsequent work, they combine their library approach with an LSTM model, training the model on promising sequences selected from the library, to design new humanized and developable nanobodies (93).

These library approaches show that there is potential to achieve multi-parameter optimization provided there is access to high-throughput and high-quality data for model training. However, if we are to address this problem computationally, the necessity for robust validation methods becomes even greater, since the entanglement of different antibody properties will make assessment ever more complex. In some cases, the complex interplay between developability properties may even render complete multi-parameter optimization impossible to achieve.

## Discussion

Humanization is the leading technique for mitigating the immunogenicity associated with therapeutics derived from non-human sources. Primarily, this has been achieved using traditional experimental strategies such as chimerization and CDR loop grafting, alongside necessary back mutations. Nonetheless, the increased availability of sequence and structural data has now facilitated the development of machine learning-based tools for humanness scoring and humanization, which suggest case-by-case engineering strategies for each input variable region sequence and are beginning to yield promising results. While most efforts are directed toward antibody humanization, the growing interest in nanobodies and their distinct structural characteristics necessitates the consideration of humanization tools tailored for this alternative scaffold. However, these efforts are hampered by the fact that nanobody data are currently relatively limited and dispersed (**Table 2**, 4, 54, 55, 94). For the plethora of alternative engineered formats now available, including bispecific or multi-specific antibodies, or those with enhanced effector functions, such as an scFv-Fc, these challenges are only enhanced and currently remain understudied (95, 96).

If *in silico* humanization tools are to be routinely implemented into therapeutic design pipelines, they must be thoroughly validated, such that computationally humanized variants can be considered confidently de-risked candidates. However, an additional layer of complexity is added since validation methods themselves vary in their reliability and proximity to the overarching phenotype of immunogenicity. Furthermore, the sensitivity of direct measures of immunogenicity such as ADA detection assays has increased over time, reflected in the surprisingly high numbers of ADAs recorded in the placebo arms of recent nanobody clinical trials (**Table 2**). This complicates the use of aggregate ADA data over the past 40 years as a benchmark and perhaps contributes to the relatively weak correlations observed between humanness metrics and the recorded abundance of ADAs (97). Even successful transition of a drug beyond Phase-I clinical trials has its limitations as a metric, due to the differential toleration of ADAs based on the severity of the indication. A better understanding of the factors underlying ADA production may be gained through investigation of healthy or immunocompromised humanized mouse immune systems (98).

Broader issues emerge across the full landscape of antibody properties, where the successful humanization of candidates might inadvertently compromise other developability attributes. These can often be addressed successfully during formulation, but formulation choices can equally yield their own developability challenges, such as anti-polyethylene glycol antibodies induced by recent biopharmaceuticals including modular mRNA vaccine technologies (99, 100). Consequently, there are growing attempts to address this multi-parameter optimization problem computationally, leveraging machine-learning techniques and the greater data availability, though these fall prey to the same need for more routine and systematic experimental validation.

More generally, the field at present functions on a certain definition of humanness. This definition is challenged by the fact that apparently genetically human antibodies can still provoke ADA production (1). This is unsurprising due to the genetic diversity sampled across humans, perhaps most pronounced in our highly personalized sets of HLA alleles that influence T cell-assisted B cell activation. Moreover, it remains hard to robustly capture the humanness of non-germline regions such as the recombination junctions or of randomness introduced by somatic hypermutation. Particularly, we highlight the emergence of machine-learning methods in antibody humanization, built using resources such as OAS (54, 55). There is an urgent need for wider representation in the available repertoire sequence datasets, since any tools trained on them will inherit their underlying biases (101). Ensuring this heterogeneity will be crucial toward accurately quantifying humanness and creating computationally designed therapeutics that exhibit general compatibility across populations.

## Author contributions

GG: Writing – original draft, Writing – review & editing. MR: Supervision, Writing – review & editing. AW: Data curation, Writing – review & editing. CD: Supervision, Writing – review & editing.

## References

[1] Harding FA Stickler MM Razo J DuBridge RB. The immunogenicity of humanized and fully human antibodies: residual immunogenicity resides in the CDR regions. MAbs. (2010) 2:256–65. doi: 10.4161/mabs.2.3.11641 PMC2881252 20400861

[2] Hansel TT Kropshofer H Singer T Mitchell JA George AJ. The safety and side effects of monoclonal antibodies. Nat Rev Drug Discovery. (2010) 9:325–38. doi: 10.1038/nrd3003 20305665

[3] Lu R-M Hwang Y-C Liu IJ Lee C-C Tsai H-Z Li H-J. Development of therapeutic antibodies for the treatment of diseases. J Biomed Science. (2020) 27:1. doi: 10.1186/s12929-019-0592-z PMC6939334 31894001

[4] Raybould MIJ Marks C Lewis AP Shi J Bujotzek A Taddese B. Thera-SAbDab: the therapeutic structural antibody database. Nucleic Acids Res. (2020) 48:D383–d8. doi: 10.1093/nar/gkz827 PMC6943036 31555805

[5] Kim J McFee M Fang Q Abdin O Kim PM. Computational and artificial intelligence-based methods for antibody development. Trends Pharmacol Sci. (2023) 44:175–89. doi: 10.1016/j.tips.2022.12.005 36669976

[6] Wesolowski J Alzogaray V Reyelt J Unger M Juarez K Urrutia M. Single domain antibodies: promising experimental and therapeutic tools in infection and immunity. Med Microbiol Immunol. (2009) 198:157–74. doi: 10.1007/s00430-009-0116-7 PMC2714450 19529959

[7] Rossotti MA Bélanger K Henry KA Tanha J. Immunogenicity and humanization of single-domain antibodies. FEBS J. (2022) 289:4304–27. doi: 10.1111/febs.15809 33751827

[8] Krah S Schröter C Zielonka S Empting M Valldorf B Kolmar H. Single-domain antibodies for biomedical applications. Immunopharmacol Immunotoxicol. (2016) 38:21–8. doi: 10.3109/08923973.2015.1102934 26551147

[9] Vincke C Loris R Saerens D Martinez-Rodriguez S Muyldermans S Conrath K. General strategy to humanize a camelid single-domain antibody and identification of a universal humanized nanobody scaffold. J Biol Chem. (2009) 284:3273–84. doi: 10.1074/jbc.M806889200 19010777

[10] Czajka TF Vance DJ Mantis NJ. Slaying SARS-coV-2 one (Single-domain) antibody at a time. Trends Microbiol. (2021) 29:195–203. doi: 10.1016/j.tim.2020.12.006 33446406 PMC7744031

[11] Bannas P Hambach J Koch-Nolte F. Nanobodies and nanobody-based human heavy chain antibodies as antitumor therapeutics. Front Immunol. (2017) 8:1603. doi: 10.3389/fimmu.2017.01603 29213270 PMC5702627

[12] Zavrtanik U Lukan J Loris R Lah J Hadži S. Structural basis of epitope recognition by heavy-chain camelid antibodies. J Mol Biol. (2018) 430:4369–86. doi: 10.1016/j.jmb.2018.09.002 30205092

[13] Muyldermans S. Nanobodies: natural single-domain antibodies. Annu Rev Biochem. (2013) 82:775–97. doi: 10.1146/annurev-biochem-063011-092449 23495938

[14] Gordon GL Capel HL Guloglu B Richardson E Stafford RL Deane CM. A comparison of the binding sites of antibodies and single-domain antibodies. Front Immunol. (2023) 14:1231623. doi: 10.3389/fimmu.2023.1231623 37533864 PMC10392943

[15] Almagro JC Fransson J. Humanization of antibodies. Front Biosci. (2008) 13:1619–33. doi: 10.2741/2786 17981654

[16] Safdari Y Farajnia S Asgharzadeh M Khalili M. Antibody humanization methods - a review and update. Biotechnol Genet Eng Rev. (2013) 29:175–86. doi: 10.1080/02648725.2013.801235 24568279

[17] Norman RA Ambrosetti F Bonvin A Colwell LJ Kelm S Kumar S. Computational approaches to therapeutic antibody design: established methods and emerging trends. Brief Bioinform. (2020) 21:1549–67. doi: 10.1093/bib/bbz095 PMC7947987 31626279

[18] Wilman W Wróbel S Bielska W Deszynski P Dudzic P Jaszczyszyn I. Machine-designed biotherapeutics: opportunities, feasibility and advantages of deep learning in computational antibody discovery. Briefings Bioinf. (2022) 23:bbac267. doi: 10.1093/bib/bbac267 PMC9294429 35830864

[19] Akbar R Bashour H Rawat P Robert PA Smorodina E Cotet TS. Progress and challenges for the machine learning-based design of fit-for-purpose monoclonal antibodies. MAbs. (2022) 14:2008790. doi: 10.1080/19420862.2021.2008790 35293269 PMC8928824

[20] Wang B Gallolu Kankanamalage S Dong J Liu Y. Optimization of therapeutic antibodies. Antibody Ther. (2021) 4:45–54. doi: 10.1093/abt/tbab003 PMC7944496 33928235

[21] Zhou Y Huang Z Li W Wei J Jiang Q Yang W. Deep learning in preclinical antibody drug discovery and development. Methods. (2023) 218:57–71. doi: 10.1016/j.ymeth.2023.07.003 37454742

[22] Makowski EK Wu L Gupta P Tessier PM. Discovery-stage identification of drug-like antibodies using emerging experimental and computational methods. MAbs. (2021) 13:1895540. doi: 10.1080/19420862.2021.1895540 34313532 PMC8346245

[23] Khetan R Curtis R Deane CM Hadsund JT Kar U Krawczyk K. Current advances in biopharmaceutical informatics: guidelines, impact and challenges in the computational developability assessment of antibody therapeutics. MAbs. (2022) 14:2020082. doi: 10.1080/19420862.2021.2020082 35104168 PMC8812776

[24] Evers A Malhotra S Sood VD eds. In Silico Approaches to Deliver Better Antibodies by Design: The Past, the Present and the Future (2023) [preprint].

[25] Abhinandan KR Martin AC. Analyzing the “degree of humanness” of antibody sequences. J Mol Biol. (2007) 369:852–62. doi: 10.1016/j.jmb.2007.02.100 17442342

[26] Thullier P Huish O Pelat T Martin AC. The humanness of macaque antibody sequences. J Mol Biol. (2010) 396:1439–50. doi: 10.1016/j.jmb.2009.12.041 20043919

[27] Gao SH Huang K Tu H Adler AS. Monoclonal antibody humanness score and its applications. BMC Biotechnol. (2013) 13:55. doi: 10.1186/1472-6750-13-55 23826749 PMC3729710

[28] Lazar GA Desjarlais JR Jacinto J Karki S Hammond PW. A molecular immunology approach to antibody humanization and functional optimization. Mol Immunol. (2007) 44:1986–98. doi: 10.1016/j.molimm.2006.09.029 17079018

[29] Seeliger D. Development of scoring functions for antibody sequence assessment and optimization. PloS One. (2013) 8:e76909. doi: 10.1371/journal.pone.0076909 24204701 PMC3804498

[30] Clavero-Álvarez A Di Mambro T Perez-Gaviro S Magnani M Bruscolini P. Humanization of antibodies using a statistical inference approach. Sci Rep. (2018) 8:14820. doi: 10.1038/s41598-018-32986-y 30287940 PMC6172228

[31] Schmitz S Soto C Crowe JE Jr. Meiler J. Human-likeness of antibody biologics determined by back-translation and comparison with large antibody variable gene repertoires. MAbs. (2020) 12:1758291. doi: 10.1080/19420862.2020.1758291 32397786 PMC8648325

[32] Marks C Hummer AM Chin M Deane CM. Humanization of antibodies using a machine learning approach on large-scale repertoire data. Bioinformatics. (2021) 37:4041–7. doi: 10.1093/bioinformatics/btab434 PMC8760955 34110413

[33] Prihoda D Maamary J Waight A Juan V Fayadat-Dilman L Svozil D. BioPhi: A platform for antibody design, humanization, and humanness evaluation based on natural antibody repertoires and deep learning. MAbs. (2022) 14:2020203. doi: 10.1080/19420862.2021.2020203 35133949 PMC8837241

[34] Uçar T Ramon A Oglic D Croasdale-wood R Diethe T Sormanni P. Improving antibody humanness prediction using patent data. ArXiv. (2024). abs/2401.14442. doi: 10.48550/arXiv.2401.14442

[35] Zou H Yuan R Lai B Dou Y Wei L Xu J. Antibody humanization via protein language model and neighbor retrieval. bioRxiv. (2023). doi: 10.1101/2023.09.04.556278

[36] Wollacott AM Xue C Qin Q Hua J Bohnuud T Viswanathan K. Quantifying the nativeness of antibody sequences using long short-term memory networks. Protein Engineering Design Selection. (2019) 32:347–54. doi: 10.1093/protein/gzz031 PMC7372931 31504835

[37] Vashchenko D Nguyen S Goncalves A FLd S Petersen B Desautels T. AbBERT: learning antibody humanness via masked language modeling. bioRxiv. (2022). doi: 10.1101/2022.08.02.502236

[38] Choi Y Hua C Sentman CL Ackerman ME Bailey-Kellogg C. Antibody humanization by structure-based computational protein design. MAbs. (2015) 7:1045–57. doi: 10.1080/19420862.2015.1076600 PMC5045135 26252731

[39] Choi Y Ndong C Griswold KE Bailey-Kellogg C. Computationally driven antibody engineering enables simultaneous humanization and thermostabilization. Protein Engineering Design Selection. (2016) 29:419–26. doi: 10.1093/protein/gzw024 PMC5036863 27334453

[40] Hsieh Y-C J-m L Chuang K-H Ho K-W Hong S-T Liu H-J. A universal in silico V(D)J recombination strategy for developing humanized monoclonal antibodies. J Nanobiotechnology. (2022) 20:58. doi: 10.1186/s12951-022-01259-2 35101043 PMC8805405

[41] Tennenhouse A Khmelnitsky L Khalaila R Yeshaya N Noronha A Lindzen M. Computational optimization of antibody humanness and stability by systematic energy-based ranking. Nat Biomed Engineering. (2024) 8:30–44. doi: 10.1038/s41551-023-01079-1 PMC10842793 37550425

[42] Sang Z Xiang Y Bahar I Shi Y. Llamanade: An open-source computational pipeline for robust nanobody humanization. Structure. (2022) 30:418–29.e3. doi: 10.1016/j.str.2021.11.006 34895471 PMC11698024

[43] Ramon A Ali M Atkinson M Saturnino A Didi K Visentin C. Assessing antibody and nanobody nativeness for hit selection and humanization with AbNatiV. Nat Mach Intelligence. (2024) 6:74–91. doi: 10.1038/s42256-023-00778-3

[44] Morrison SL Johnson MJ Herzenberg LA Oi VT. Chimeric human antibody molecules: mouse antigen-binding domains with human constant region domains. Proc Natl Acad Sci U S A. (1984) 81:6851–5. doi: 10.1073/pnas.81.21.6851 PMC392030 6436822

[45] Jones PT Dear PH Foote J Neuberger MS Winter G. Replacing the complementarity-determining regions in a human antibody with those from a mouse. Nature. (1986) 321:522–5. doi: 10.1038/321522a0 3713831

[46] Baca M Presta LG O’Connor SJ Wells JA. Antibody humanization using monovalent phage display. J Biol Chem. (1997) 272:10678–84. doi: 10.1074/jbc.272.16.10678 9099717

[47] De Pascalis R Iwahashi M Tamura M Padlan EA Gonzales NR Santos AD. Grafting of “abbreviated” complementarity-determining regions containing specificity-determining residues essential for ligand contact to engineer a less immunogenic humanized monoclonal antibody. J Immunol. (2002) 169:3076–84. doi: 10.4049/jimmunol.169.6.3076 12218124

[48] Kashmiri SV De Pascalis R Gonzales NR Schlom J. SDR grafting–a new approach to antibody humanization. Methods. (2005) 36:25–34. doi: 10.1016/j.ymeth.2005.01.003 15848072

[49] Apgar JR Mader M Agostinelli R Benard S Bialek P Johnson M. Beyond CDR-grafting: Structure-guided humanization of framework and CDR regions of an anti-myostatin antibody. MAbs. (2016) 8:1302–18. doi: 10.1080/19420862.2016.1215786 PMC5058614 27625211

[50] Dall’Acqua WF Damschroder MM Zhang J Woods RM Widjaja L Yu J. Antibody humanization by framework shuffling. Methods. (2005) 36:43–60. doi: 10.1016/j.ymeth.2005.01.005 15848074

[51] Padlan EA. A possible procedure for reducing the immunogenicity of antibody variable domains while preserving their ligand-binding properties. Mol Immunol. (1991) 28:489–98. doi: 10.1016/0161-5890(91)90163-E 1905784

[52] Roguska MA Pedersen JT Keddy CA Henry AH Searle SJ Lambert JM. Humanization of murine monoclonal antibodies through variable domain resurfacing. Proc Natl Acad Sci U S A. (1994) 91:969–73. doi: 10.1073/pnas.91.3.969 PMC521435 8302875

[53] Petersen BM Ulmer SA Rhodes ER Gutierrez-Gonzalez MF Dekosky BJ Sprenger KG. Regulatory approved monoclonal antibodies contain framework mutations predicted from human antibody repertoires. Front Immunol. (2021) 12:728694. doi: 10.3389/fimmu.2021.728694 34646268 PMC8503325

[54] Kovaltsuk A Leem J Kelm S Snowden J Deane CM Krawczyk K. Observed antibody space: A resource for data mining next-generation sequencing of antibody repertoires. J Immunol. (2018) 201:2502–9. doi: 10.4049/jimmunol.1800708 30217829

[55] Olsen TH Boyles F Deane CM. Observed Antibody Space: A diverse database of cleaned, annotated, and translated unpaired and paired antibody sequences. Protein Sci. (2022) 31:141–6. doi: 10.1002/pro.4205 PMC8740823 34655133

[56] Ackaert C Smiejkowska N Xavier C Sterckx YGJ Denies S Stijlemans B. Immunogenicity risk profile of nanobodies. Front Immunol. (2021) 12:632687. doi: 10.3389/fimmu.2021.632687 33767701 PMC7985456

[57] Keyaerts M Xavier C Heemskerk J Devoogdt N Everaert H Ackaert C. Phase I study of 68Ga-HER2-nanobody for PET/CT assessment of HER2 expression in breast carcinoma. J Nucl Med. (2016) 57:27–33. doi: 10.2967/jnumed.115.162024 26449837

[58] Ablynx aSc. A Phase II Multicenter, Randomized, Double- blind, Placebo Controlled, Dose-range Finding Study to Evaluate the Safety and Efficacy of ALX-0061 Administered Subcutaneously in Subjects With Moderate to Severe Active Systemic Lupus Erythematosus. clinicaltrials.gov (2019).

[59] Ablynx aSc. A Phase IIb Multicenter, Randomized, Double- blind Study of ALX-0061 Administered Subcutaneously as Monotherapy, in Subjects With Moderate to Severe Rheumatoid Arthritis Who Are Intolerant to Methotrexate or for Whom Continued Methotrexate Treatment is Inappropriate. clinicaltrials.gov (2019).

[60] Holz J-B Sargentini-Maier L Bruyn SD Gachályi B Udvaros I Rojkovich B. OP0043 twenty- four weeks of treatment with a novel anti-IL-6 receptor nanobody^®^ (ALX- 0061) resulted in 84% ACR20 improvement and 58% DAS28 remission in a phase I/ii study in RA. Ann Rheumatic Dis. (2013) 72:A64–4. doi: 10.1136/annrheumdis-2013-eular.248

[61] Van Roy M Ververken C Beirnaert E Hoefman S Kolkman J Vierboom M. The preclinical pharmacology of the high affinity anti-IL-6R Nanobody^®^ ALX-0061 supports its clinical development in rheumatoid arthritis. Arthritis Res Ther. (2015) 17:135. doi: 10.1186/s13075-015-0651-0 25994180 PMC4476083

[62] Bartunek J Barbato E Heyndrickx G Vanderheyden M Wijns W Holz J-B. Novel antiplatelet agents: ALX-0081, a Nanobody directed towards von Willebrand factor. J Cardiovasc Transl Res. (2013) 6:355–63. doi: 10.1007/s12265-012-9435-y 23307200

[63] Peyvandi F Scully M Kremer Hovinga JA Knöbl P Cataland S De Beuf K. Caplacizumab reduces the frequency of major thromboembolic events, exacerbations and death in patients with acquired thrombotic thrombocytopenic purpura. J Thromb Haemost. (2017) 15:1448–52. doi: 10.1111/jth.13716 28445600

[64] Scully M Cataland SR Peyvandi F Coppo P Knöbl P Kremer Hovinga JA. Caplacizumab treatment for acquired thrombotic thrombocytopenic purpura. N Engl J Med. (2019) 380:335–46. doi: 10.1056/NEJMoa1806311 30625070

[65] Schoen P Jacobs S Verschueren K Ottevaere I Sobry S Holz J-B. Anti-RANKL nanobody ALX-0141 shows sustained biomarker inhibition in a Phase I study in healthy postmenopausal Women. Bone Abstracts. (2013). Presented at the European Calcified Tissue Society Congress 2013, Bioscientifica. 1:PP135 doi: 10.1530/boneabs.1.PP135

[66] Detalle L Stohr T Palomo C Piedra PA Gilbert BE Mas V. Generation and characterization of ALX-0171, a potent novel therapeutic nanobody for the treatment of respiratory syncytial virus infection. Antimicrobial Agents Chemotherapy. (2015) 60:6–13. doi: 10.1128/AAC.01802-15 26438495 PMC4704182

[67] Cunningham S Piedra PA Martinon-Torres F Szymanski H Brackeva B Dombrecht E. Nebulised ALX-0171 for respiratory syncytial virus lower respiratory tract infection in hospitalised children: a double-blind, randomised, placebo-controlled, phase 2b trial. Lancet Respir Med. (2021) 9:21–32. doi: 10.1016/S2213-2600(20)30320-9 33002427

[68] Merck KGaA D, Germany. Multicenter, Phase I, Randomized, Double-Blind, Placebo-Controlled Trial to Assess the Safety, Tolerability, Immunogenicity, Pharmacokinetics, Pharmacodynamics and Efficacy of Multiple Ascending Doses of Subcutaneous MSB0010841 (Anti-IL17A/F Nanobody) in Male and Female Subjects With Moderate to Severe Psoriasis (Clinical trial registration No. results/NCT02156466). clinicaltrials.gov (2016).

[69] Ablynx aSc. An Open-Label Extension Study To Assess The Long-term Safety And Tolerability Of ATN-103 In Subjects With Rheumatoid Arthritis (Clinical trial registration No. NCT01063803). clinicaltrials.gov (2016).

[70] Ablynx aSc. A Randomized, Multicenter, Double-Blind, Placebo-Controlled, Multiple Ascending Dose Study Of The Safety, Tolerability, Pharmacokinetics, Pharmacodynamics, And Clinical Efficacy Of ATN-103 Administered To Japanese Subjects With Active Rheumatoid Arthritis On A Background Of Methotrexate (Clinical trial registration No. NCT01007175). clinicaltrials.gov (2013).

[71] Ablynx aSc. A Seamless, Phase 1/2, Multiple Ascending Dose, Proof Of Concept Study Of ATN-103 Administered To Subjects With Active Rheumatoid Arthritis On A Background Of Methotrexate (Clinical trial registration No. NCT00959036). clinicaltrials.gov (2013).

[72] Buyse M-A Hermans G Lindemann S Guehring H Guenther R Kellner R. Adamts binding immunoglobulins. Patent number: WO2018220234A1 (2017).

[73] Papadopoulos KP Isaacs R Bilic S Kentsch K Huet HA Hofmann M. Unexpected hepatotoxicity in a phase I study of TAS266, a novel tetravalent agonistic Nanobody^®^ targeting the DR5 receptor. Cancer Chemother Pharmacol. (2015) 75:887–95. doi: 10.1007/s00280-015-2712-0 25721064

[74] Sulea T. Humanization of camelid single-domain antibodies. Methods Mol Biol. (2022) 2446:299–312. doi: 10.1007/978-1-0716-2075-5_14 35157279

[75] Conrath K Vincke C Stijlemans B Schymkowitz J Decanniere K Wyns L. Antigen binding and solubility effects upon the veneering of a camel VHH in framework-2 to mimic a VH. J Mol Biol. (2005) 350:112–25. doi: 10.1016/j.jmb.2005.04.050 15913651

[76] Gentiluomo L Roessner D Streicher W Mahapatra S Harris P Frieß W. Characterization of native reversible self-association of a monoclonal antibody mediated by fab-fab interaction. J Pharm Sci. (2020) 109:443–51. doi: 10.1016/j.xphs.2019.09.021 31563513

[77] Voynov V Chennamsetty N Kayser V Helk B Trout BL. Predictive tools for stabilization of therapeutic proteins. MAbs. (2009) 1:580–2. doi: 10.4161/mabs.1.6.9773 PMC2791315 20068399

[78] Waibl F Fernández-Quintero ML Kamenik AS Kraml J Hofer F Kettenberger H. Conformational ensembles of antibodies determine their hydrophobicity. Biophys J. (2021) 120:143–57. doi: 10.1016/j.bpj.2020.11.010 PMC7820740 33220303

[79] Mitchell LS Colwell LJ. Comparative analysis of nanobody sequence and structure data. Proteins. (2018) 86:697–706. doi: 10.1002/prot.25497 29569425 PMC6033041

[80] Kelow SP Adolf-Bryfogle J Dunbrack RL. Hiding in plain sight: structure and sequence analysis reveals the importance of the antibody DE loop for antibody-antigen binding. MAbs. (2020) 12:1840005. doi: 10.1080/19420862.2020.1840005 33180672 PMC7671036

[81] Hummer AM Deane CM. Designing stable humanized antibodies. Nat Biomed Engineering. (2024) 8:3–4. doi: 10.1038/s41551-023-01168-1 38151639

[82] Abanades B Wong WK Boyles F Georges G Bujotzek A Deane CM. ImmuneBuilder: Deep-Learning models for predicting the structures of immune proteins. Commun Biol. (2023) 6:575. doi: 10.1038/s42003-023-04927-7 37248282 PMC10227038

[83] Jain T Boland T Vásquez M. Identifying developability risks for clinical progression of antibodies using high-throughput in *vitro* and in silico approaches. MAbs. (2023) 15:2200540. doi: 10.1080/19420862.2023.2200540 37072706 PMC10114995

[84] Heads JT Kelm S Tyson K Lawson ADG. A computational method for predicting the aggregation propensity of IgG1 and IgG4(P) mAbs in common storage buffers. MAbs. (2022) 14:2138092. doi: 10.1080/19420862.2022.2138092 36418193 PMC9704409

[85] Zhang W Wang H Feng N Li Y Gu J Wang Z. Developability assessment at early-stage discovery to enable development of antibody-derived therapeutics. Antib Ther. (2023) 6:13–29. doi: 10.1093/abt/tbac029 36683767 PMC9847343

[86] Vaisman-Mentesh A Gutierrez-Gonzalez M DeKosky BJ Wine Y. The molecular mechanisms that underlie the immune biology of anti-drug antibody formation following treatment with monoclonal antibodies. Front Immunol. (2020) 11:1951. doi: 10.3389/fimmu.2020.01951 33013848 PMC7461797

[87] Reynisson B Alvarez B Paul S Peters B Nielsen M. NetMHCpan-4.1 and NetMHCIIpan-4.0: improved predictions of MHC antigen presentation by concurrent motif deconvolution and integration of MS MHC eluted ligand data. Nucleic Acids Res. (2020) 48:W449–W54. doi: 10.1093/nar/gkaa379 PMC7319546 32406916

[88] Makowski EK Kinnunen PC Huang J Wu L Smith MD Wang T. Co-optimization of therapeutic antibody affinity and specificity using machine learning models that generalize to novel mutational space. Nat Commun. (2022) 13:3788. doi: 10.1038/s41467-022-31457-3 35778381 PMC9249733

[89] Bachas S Rakocevic G Spencer D Sastry AV Haile R Sutton JM. Antibody optimization enabled by artificial intelligence predictions of binding affinity and naturalness. bioRxiv. (2022). doi: 10.1101/2022.08.16.504181

[90] Liu G Zeng H Mueller J Carter B Wang Z Schilz J. Antibody complementarity determining region design using high-capacity machine learning. Bioinformatics. (2020) 36:2126–33. doi: 10.1093/bioinformatics/btz895 PMC7141872 31778140

[91] Saka K Kakuzaki T Metsugi S Kashiwagi D Yoshida K Wada M. Antibody design using LSTM based deep generative model from phage display library for affinity maturation. Sci Rep. (2021) 11:5852. doi: 10.1038/s41598-021-85274-7 33712669 PMC7955064

[92] Arras P Yoo HB Pekar L Schröter C Clarke T Krah S. A library approach for the *de novo* high-throughput isolation of humanized VHH domains with favorable developability properties following camelid immunization. MAbs. (2023) 15:2261149. doi: 10.1080/19420862.2023.2261149 37766540 PMC10540653

[93] Arras P Yoo HB Pekar L Clarke T Friedrich L Schröter C. AI/ML combined with next-generation sequencing of VHH immune repertoires enables the rapid identification of *de novo* humanized and sequence-optimized single domain antibodies: a prospective case study. Front Mol Biosci. (2023) 10:1249247. doi: 10.3389/fmolb.2023.1249247 37842638 PMC10575757

[94] Raybould MIJ Kovaltsuk A Marks C Deane CM. CoV-AbDab: the coronavirus antibody database. Bioinformatics. (2020) 37:734–5. doi: 10.1093/bioinformatics/btaa739 PMC7558925 32805021

[95] Brinkmann U Kontermann RE. The making of bispecific antibodies. MAbs. (2017) 9:182–212. doi: 10.1080/19420862.2016.1268307 28071970 PMC5297537

[96] Keri D Walker M Singh I Nishikawa K Garces F. Next generation of multispecific antibody engineering. Antibody Ther. (2023) 7:37–52. doi: 10.1093/abt/tbad027 PMC10791046 38235376

[97] Song S Yang L Trepicchio WL Wyant T. Understanding the supersensitive anti-drug antibody assay: unexpected high anti-drug antibody incidence and its clinical relevance. J Immunol Res. (2016) 2016:3072586. doi: 10.1155/2016/3072586 27340678 PMC4906211

[98] Chen J Liao S Xiao Z Pan Q Wang X Shen K. The development and improvement of immunodeficient mice and humanized immune system mouse models. Front Immunol. (2022) 13:1007579. doi: 10.3389/fimmu.2022.1007579 36341323 PMC9626807

[99] Kozma GT Shimizu T Ishida T Szebeni J. Anti-PEG antibodies: Properties, formation, testing and role in adverse immune reactions to PEGylated nano-biopharmaceuticals. Adv Drug Delivery Rev. (2020) 154–155:163–75. doi: 10.1016/j.addr.2020.07.024 32745496

[100] Ju Y Carreño JM Simon V Dawson K Krammer F Kent SJ. Impact of anti-PEG antibodies induced by SARS-CoV-2 mRNA vaccines. Nat Rev Immunol. (2023) 23:135–6. doi: 10.1038/s41577-022-00825-x PMC9764299 36539526

[101] Olsen TH Moal IH Deane CM. Addressing the antibody germline bias and its effect on language models for improved antibody design. bioRxiv. (2024). doi: 10.1101/2024.02.02.578678 PMC11543624 39460949

